# A Thiazole Orange Derivative Targeting the Bacterial Protein FtsZ Shows Potent Antibacterial Activity

**DOI:** 10.3389/fmicb.2017.00855

**Published:** 2017-05-11

**Authors:** Ning Sun, Yu-Jing Lu, Fung-Yi Chan, Ruo-Lan Du, Yuan-yuan Zheng, Kun Zhang, Lok-Yan So, Ruben Abagyan, Chao Zhuo, Yun-Chung Leung, Kwok-Yin Wong

**Affiliations:** ^1^Department of Applied Biology and Chemical Technology and the State Key Laboratory of Chirosciences, The Hong Kong Polytechnic UniversityHong Kong, Hong Kong; ^2^Institute of Natural Medicine and Green Chemistry, School of Chemical Engineering and Light Industry, Guangdong University of TechnologyGuangzhou, China; ^3^Skaggs School of Pharmacy and Pharmaceutical Sciences, University of California, San Diego, La JollaCA, USA; ^4^State Key Laboratory of Respiratory Diseases, the First Affiliated Hospital of Guangzhou Medical UniversityGuangzhou, China

**Keywords:** bacterial resistance, antibacterial activity, cell division, FtsZ inhibitor, FtsZ polymerization

## Abstract

The prevalence of multidrug resistance among clinically significant bacteria calls for the urgent development of new antibiotics with novel mechanisms of action. In this study, a new small molecule exhibiting excellent inhibition of bacterial cell division with potent antibacterial activity was discovered through cell-based screening. The compound exhibits a broad spectrum of bactericidal activity, including the methicillin-resistant *Staphylococcus aureus*, vancomycin-resistant *Enterococcus* and NDM-1 *Escherichia coli*. The *in vitro* and *in vivo* results suggested that this compound disrupts the dynamic assembly of FtsZ protein and Z-ring formation through stimulating FtsZ polymerization. Moreover, this compound exhibits no activity on mammalian tubulin polymerization and shows low cytotoxicity on mammalian cells. Taken together, these findings could provide a new chemotype for development of antibacterials with FtsZ as the target.

## Introduction

Treatment of antibiotic-resistant bacterial infections is becoming more difficult because bacteria develop resistance to conventional antibiotics at an alarming speed (Payne et al., 2007; Arias and Murray, 2009; Silver, 2011; Wright, 2012). Methicillin-resistant *Staphylococcus aureus* (MRSA) and vancomycin-resistant *Enterococcus faecium* (VREF) are typical examples of Gram-positive bacteria which have already shown resistance to the wildly prescribed antibiotics including methicillin and vancomycin (Gould et al., 2012; Humphries et al., 2013). This situation is also alarming for Gram-negative bacteria. The World Health Organization (WHO) has just released a list of the drug-resistant bacteria which new antibiotics are desperately needed. In this list, carbapenem resistant Gram-negative organisms are in the critical priority (Willyard, 2017). In addition, Superbugs with New Delhi metallo-beta-lactamase-1 (NDM-1) are known to be highly resistant to most antibiotics and only tigecycline and colistin are still effective nowadays (Kumarasamy et al., 2010; Walsh et al., 2011). Therefore, new types of antibacterial agents with new molecular scaffolds and mechanisms of action are urgently needed (Devasahayam et al., 2010; Wright, 2012).

Understanding bacterial cell division is believed to be critical in new antibiotic development because cell division is an essential process for bacterial survival and the bacterial divisome possesses a complex set of biochemical machinery that contains many proteins as potential drug targets. Among these proteins, filamenting temperature-sensitive mutant Z (FtsZ) has been identified as a very critical protein that can influence bacterial cell division and it is highly conserved in a wide range of bacteria (Erickson, 1995, 1997; Margolin, 2000; Addinall and Holland, 2002). During bacterial cytokinesis, FtsZ assembles into a highly dynamic cytoskeleton scaffold (the Z-ring) by undergoing GTP-dependent polymerization, generating head-to-tail protofilaments and assembling into bundles at the site of septum formation (Bi and Lutkenhaus, 1991; Oliva et al., 2004; Li et al., 2013). Subsequently, FtsZ recruits other downstream proteins responsible for the invagination of cell membrane and septum formation, completing the bacterial cell division (Margolin, 2005; Adams and Errington, 2009).

The high conservation and functional importance among antibiotic-sensitive and antibiotic-resistant bacteria established FtsZ as an attractive target for the development of new therapeutic agents. In recent years, a number of small molecule inhibitors of FtsZ have already been revealed to perturb FtsZ polymerization and inhibit bacterial cell division (Bierer et al., 1998; Beuria et al., 2005; Schaffner-Barbero et al., 2012; Li et al., 2015; Haranahalli et al., 2016; Hurley et al., 2016; Qiang et al., 2016; Bi et al., 2017). These studies suggest that the molecules impair bacterial growth through disrupting the dynamic assembly or/and GTP hydrolysis of FtsZ. Regarding synthetic inhibitors, PC190723 is the most studied compound so far (Haydon et al., 2008, 2010; Andreu et al., 2010; Adams et al., 2011; Elsen et al., 2012; Tan et al., 2012). This difluorobenzamide derivative enhances FtsZ polymerization and exhibits potent antibacterial activity [e.g., MIC (MRSA) = 1 μg/mL], but exerts little effect on gram-negative strains (Haydon et al., 2008). In order to improve the pharmacological properties of PC190723, Kaul et al. (2013a,b, 2015) designed and synthesized some prodrugs of PC190723, these drug candidates showed superior antibacterial potencies and improved pharmacokinetic profiles compared with PC190723. Among the natural products, berberine and its 9-phenoxyalkylberberine derivatives block the protein assembly and inhibit the GTPase of FtsZ (Domadia et al., 2008; Sun et al., 2014). Compounds from these derivatives exhibited strong antibacterial actives against MRSA and VREF [MIC values = 2–8 μg/mL]. These berberine analogs also showed a moderate inhibition on the growth of Gram-negative bacteria such as *E. coli* and *K. pneumoniae* with MIC values of 32–128 μg/mL (Sun et al., 2014). Last but not least, a few peptidic inhibitors have also been studied. Among these peptides, the cyclic octapeptides design by Pieraccini et al. (2013) via computational method showed strong perturbed effects on GTPase of FtsZ and notable inhibition of FtsZ polymerization.

**FIGURE 1:**
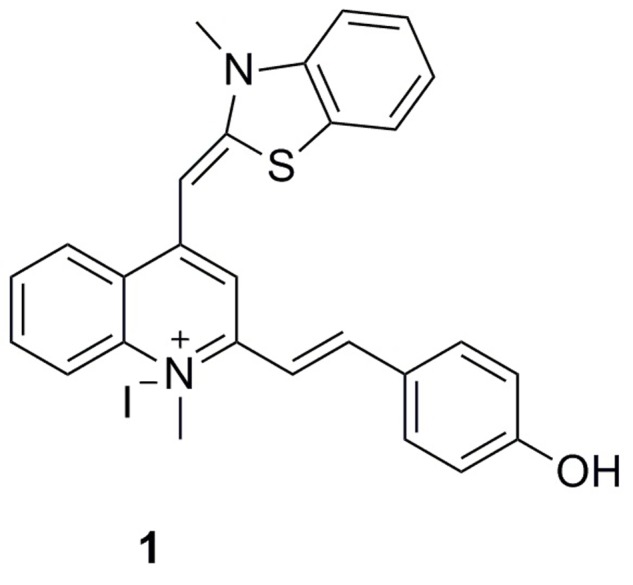
**Chemical structures of 1**.

To expand the existing chemical diversity with innovative chemotypes targeting bacterial cell division seems an opportunity. In this study, we have focused our efforts on the identification of new small molecules that block the bacterial cell division process and disrupt FtsZ activity, and successfully identified a potent cell division inhibitor, 2-((E)-4- Hydroxystyryl)-1-methyl-4-((Z)-(3-methylbenzo[d]thiazol-2(3H)-ylidene)methyl)quinolin-1-ium iodide (**1**), by cell-based screening (**Figure 1**). The results obtained suggest that this new FtsZ targeting compound inhibits bacterial cell division with high antibacterial activity.

## Materials and Methods

### Cell Division Inhibitory Screening Assay

Cell division inhibitory activity of the tested compounds was performed as described previously (Stokes et al., 2005). Overnight *B. subtilis* culture was grown in starvation medium supplemented with 1% hydrolyzed casein and then diluted in starvation medium supplemented with 3% hydrolyzed casein and grown at 37°C. The culture was diluted to A_600_ of ∼0.06, and 10 μL aliquots were added to transparent 96-well microtiter plates containing dilutions of the screening compounds in 100 μL volumes of medium. After incubation for approximately 5 h (4–5 generations) at 37°C, 20 μL culture samples were transferred to poly-L-lysine-coated slides for microscopy. Cell morphology was assessed by phase-contrast light microscopy.

### Antimicrobial Susceptibility Assay

*B. subtilis*, *S. aureus* USA300 #417 and *S. aureus* USA300 #2690 tested in this assay were already available in our in-house collection, *S. pyogenes* (ID:0716100032) and *S. agalactiae* (ID:0216100214) are clinical isolated strains in the First Affiliated Hospital of Guangzhou Medical University, other strains were purchased from American Type Culture Collection (ATCC, USA). Antimicrobial susceptibility tests were conducted in 96-well microplates using the broth microdilution procedure described in the Clinical and Laboratory Standards Institute (CLSI) guidelines (Wikler et al., 2009). Cation-adjusted Mueller Hinton broth for all the *S. aureus* strains, or Brain Heart Infusion broth for antibiotic-susceptible *E. faecium* strain ATCC 49624, vancomycin-resistant *E. faecium* strain ATCC 700221, *S. agalactiae* ATCC 13813, *S. pyogenes* ATCC 12344, *S. pyogenes* ATCC 19615 and *M. catarrhalis* ATCC 25240, or Mueller Hinton broth for the other strains were used in the assays. After incubation for 18 h at 37°C, the absorbance at 600 nm (A_600_) was recorded using a microplate reader (Bio-Rad laboratory Ltd., UK) and the percentage of bacterial cell inhibition with respect to vehicles (1% DMSO) was calculated. The MIC was defined as the lowest compound concentration at which the growth of bacteria was inhibited by ≥90%. Three independent assays were performed for each test.

### Time-Killing Curve Assay

A growing culture of *S. aureus* ATCC 29213 or *E. coli* ATCC 25922 were diluted to approximately 10^5^ CFU.mL^-1^ in volumes of Cation-adjusted Mueller Hinton broth or Mueller Hinton broth, respectively, containing various concentrations of compound **1**. Cultures were incubated at 37°C, shaking. At the appropriate time intervals, 100 μL samples were removed for serial dilution in 900 μL volumes of Mueller Hinton broth and 100 μL volumes from three dilutions were spread on to MH agar. Cell counts (CFU.ml^-1^) were enumerated after incubating the plates at 37°C for 18 h.

### Monitoring FtsZ Assembly with Light Scattering

*Staphylococcus aureus* and *E. coli* FtsZ was cloned, overexpressed, and purified as described previously (Sun et al., 2014; Li et al., 2015). The light scattering assay was performed using a protocol adapted from the literature (Andreu et al., 2010). The polymerization of recombinant *Sa*FtsZ or *Ec*FtsZ was measured using 90° light scattering in a thermostatically (37°C) controlled fluorescence spectrometer (Agilent Cary Eclipse). Both excitation and emission wavelengths were set to 600 nm with a slit width of 2.5 nm. FtsZ (6 μM) in 20 mM of Tris buffer (pH 7.4, containing 0.01% Triton X-100 to avoid compound aggregation) was placed in a 10 × 2 mm (excitation path) cell, the reaction was started by consecutive additions of 20 mM KCl, 5 mM MgCl_2_, 1 mM GTP and different concentrations of the test compound. One percentage DMSO and 20 μg/mL methicillin were tested as vehicle and negative in this assay.

### Transmission Electron Microscopy (TEM)

*Staphylococcus aureus* FtsZ (12 μM) was incubated in the absence and in the presence of 3 μg/mL of **1** in 50 mM MOPS buffer (pH 6.5) at 25°C. After 10 min, 5 mM MgCl_2_, 50 mM KCl, and 1 mM GTP were added to the reaction mixtures and incubated at 37°C for 15 min. Then, 10 μL of the sample mixtures were placed on a glow-discharged Formvar carbon-coated copper grid (400 mesh) for 10 min. The grids were subsequently subjected to negative staining using 10 μL of 0.5% phosphotungstic acid (PTA) for 30 s, air-dried and digital images of the specimen were observed with a transmission electron microscope (JEOL model JEM 2010) operated at 200 kV and equipped with a Gatan MSC 794 CCD camera.

### GTPase Activity Assay

The Effect of **1** on the GTPase activity of recombinant FtsZ was measured in 96-well microplates using a CytoPhos phosphate assay Biochem Kit (Cytoskeleton, USA) according to an optimized protocol and the manufacturer’s instructions. FtsZ (6 μM) was preincubated with vehicle (1% DMSO) or different concentrations of each test compound in 20 mM Tris buffer (pH 7.4, containing 0.01% Triton X-100 to avoid compound aggregation) for 10 min at 25°C. Then 5 mM of MgCl_2_ and 200 mM of KCl were added. Reactions were started with the addition of 500 mM GTP and incubated at 37°C. After 30 min, the reactions were quenched by adding 100 mL of Cytophos reagent for 10 min. Inorganic phosphate was quantified by measuring the absorbance at 650 nm with a microplate reader (Bio-Rad laboratory Ltd., UK).

### Monitoring Secondary Structural Changes of FtsZ

*Sa*FtsZ (10 μM) was incubated without or with **1** at different concentrations (3 and 6 μg/mL) in 20 mM Tris buffer (pH 7.4, containing 0.01% Triton X-100 to avoid compound aggregation) for 30 min at 25°C. The far-UV CD spectrum was monitored over a wavelength range of 200–250 nm using a JASCO J-810 spectropolarimeter equipped with a temperature controller and a 0.1 cm path length quartz cuvette. An average of five scans was taken for each spectrum. Deconvolution and statistical analysis of the CD spectra were performed using Jasco software and Origin 6.0 software, respectively.

### Z-Ring Visualization in *B. subtilis* Cells

A culture of *B. subtilis* containing the IPTG-inducible plasmid for the overexpression of green fluorescence protein (GFP)-tagged FtsZ was grown in LB medium supplemented with 30 μg/mL of chloramphenicol. After overnight incubation, a sample of the culture was diluted to 1% in the LB medium containing 1 μg/mL of **1** and 40 μM of IPTG. After 4 h incubation at 37°C, the *B. subtilis* cells were fixed, harvested and resuspended in PBS buffer containing 0.25% of agarose. 10 μL of sample mixture were added to a pretreated microscopic slide with 0.1% (w/v) poly-L-lysine and visualized using a fluorescence microscope at 60× oil immersion magnification with a standard FITC filter set. The images were captured using an Olympus Bio Imaging Navigator FSX 100 microscope.

### Molecular Docking Simulation of Compound 1

The molecular modeling procedures were performed using the MolSoft ICM 3.8.4 software (Neves et al., 2012). The X-ray crystal structure of *S. aureus* FtsZ in complex with a cell division inhibitor (PC190723) and GDP was downloaded from the PDB database (PDB entry: 4DXD; resolution: 2.0 Å) (Tan et al., 2012). Water molecules and co-crystal ligands were removed from the structure and the protein was prepared for docking using an automated procedure of ICM. Briefly, the hydrogen atoms were added to the structure, MMFF atom types and charges were assigned, and His, Asn and Gln residues were optimized to the best hydrogen bonding network. Energy maps accounting for hydrogen bonding, van der Waals, hydrophobic and electrostatic interactions were pre-calculated for two possible binding sites centered at either the co-crystal ligand PC190723, or at the GDP molecule. Each putative binding site was defined as a cubic grid including all FtsZ residues within a 10 Å cutoff from the co-crystal structure. The grid spacing was set to 0.5 Å. The structure of **1** was sketched in 2D and converted into 3D using the ICM molecule editor. The molecules was flexibly docked against the pre-calculated grid potential maps and scored using the ICM scoring function. One hundred independent docking runs were performed and the results were clustered in order to filter repeated binding modes. The top-scoring poses were visually inspected.

### Effects on Eukaryotic Tubulin Polymerization

The effects of **1** on eukaryotic tubulin polymerization were monitored by fluorescence microscopy via using a tubulin polymerization assay kit (BK011P, Cytoskeleton, Inc). A known tubulin polymerization enhancer (paclitaxel) and a known inhibitor of tubulin-dependent GTP hydrolysis (vinblastine) were tested as reference compounds in the same assay conditions. The concentration of **1** (20 μM) in this assay is much higher than that in the FtsZ polymerization assay, to make sure whether this compound has an effect on tubulin or not.

### Cytotoxicity Test of Compound 1

L929 mouse fibroblasts cells and HK-2 renal epithelial cells were removed from the sterile cell culture flasks with trypsin and neutralized with fetal bovine serum. After washing with phosphate buffered saline and centrifugation, cells were re-suspended in complete cell culture medium and the concentration was adjusted at approximately 1 × 10^5^ cells mL^-1^. Cells seeded in the 96 wells microtiter plates for 24 h were used for the evaluation of the tested compounds. 3-(4,5-dimethylthiazol-2-yl)-5-(3-carboxymethoxyphenyl)-2-(4-sulfophenyl)-2H-tetrazolium (MTS) was purchased from Promega (Madison, WI, USA). For the MTS assay, cells (5,000 per well) were seeded into 96-well plates. After treatment with compounds of different concentrations for 24 h, cells were added with MTS at a final concentration of 0.3 mg/mL, followed by incubation for another 2 h. The optical density (OD) of each well was determined at 490 nm (background subtraction at 690 nm) by a SpectraMax 340 microplate reader (Molecular Devices, Sunnyvale, CA, USA). The growth inhibitory ratio was calculated as follows: Growth inhibitory ratio = (A_control_ - A_sample_)/A_control_ (where A is the OD value per well).

### Antimicrobial Study of Compound 1 under Different Nutrient Conditions

To determine the growth curves of this assay, a single colony of *B. subtilis* was inoculated in 10 mL LB or 10^-2^ LB at 37°C for 24 h with shaking. The culture was harvested by centrifuging at 13,000 rpm for 5 min at 4°C, and the cells were diluted with LB or 10^-2^ LB to 0.5 McFarland standard (approximate cell density is ∼10^8^ CFU.mL^-1^). Aliquot of 10 μL 0.5 McFarland standard cultures were diluted into 10 mL LB or 10^-2^ LB containing various concentrations of compound **1**, respectively. Cultures were incubated at 37°C with shaking. At appropriate time intervals, 100 μL samples were taken from the culture solution for serial dilution in 900 μL of the cultured medium broth and 100 μL from three dilutions were spread over MH agar. Cell counts (CFU.ml^-1^) were enumerated after the plates incubated at 37°C for 18 h. For the morphology observation, *B. subtilis* cells were cultured in LB or 10^-2^ LB for 24 h without or with 0.75 μg/mL of **1**. Then 20 μL culture samples were transferred to poly-L-lysine-coated slides for microscopy. Cell morphology was assessed by phase-contrast light microscopy.

### Synthesis and Characterization of Compound 1

Refer to the Scheme S1 and Figures S3–S6 in the Electronic Supplementary Material.

## Results

### Compound 1 Induced Bacterial Cell Elongation

**FIGURE 2:**
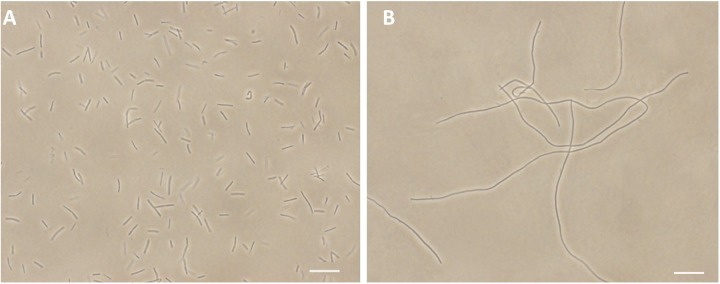
**Inhibition of cell division by 1.** Cells of *B. subtilis* 168 were grown in the absence **(A)**, and presence of 1 μg/mL of **1 (B)**. Scale Bar = 15 μm.

FtsZ targeting compounds can disrupt the cell division function of FtsZ protein, leading rod-shaped bacteria into cell elongation. Because most of the reported FtsZ inhibitors do not have or only have weak inhibitory effect against the Gram-negative strains (Haranahalli et al., 2016; Hurley et al., 2016), we therefore initiated the screen with *B. subtilis* 168 in the presence of a small compound library including 40 natural products, 20 natural product derivatives and 140 general compounds which are either commercially available or synthesized in our laboratory. The screen (Stokes et al., 2005; Li et al., 2015) generated one potential hit, 2-((E)-4-hydroxystyryl)-1-methyl-4-((Z)-(3-methylbenzo[d]thiazol-2(3H)-ylidene)methyl)quinolin-1-ium iodide (compound **1**). As shown in **Figure 2B**, **1** is able to increase the cell length of *B. subtilis* significantly at low concentration (1 μg/mL), as compared to untreated cells (**Figure 2A**), suggesting a mechanism of antibacterial-induced cell filamentation.

### Antibacterial Activity of Compound 1

We further tested the antibacterial activity of **1** against an extended panel of clinically relevant bacterial strains, including antibiotic-resistant strains. Methicillin and ceftazidime were tested under the same assay conditions as a reference compound. The results showed that **1** has potent and broad spectrum antibacterial activity against most of the Gram-positive and -negative strains which were tested, as well as all strains and species of S*taphylococci* that were tested [minimal inhibitory concentration (MIC) in the range of 0.75–3.0 μg/mL], including nine MRSA strains (**Table 1**). **1** can inhibit the growth of antibiotic-susceptible and antibiotic-resistant *S. aureus* strains with MIC values of 1.5–3 μg/mL, revealed that **1** have a better anti-*S. aureus* effect than methicillin and ceftazidime (**Table 1**). The potency of **1** was more than 100 times greater than that of methicillin against most of the MRSA strains. The growth of vancomycin-susceptible and vancomycin-resistant *E. faecium* were inhibited with MIC values of 0.75–1.5 μg/mL. For Gram-negative strains, **1** strongly inhibited the growth of *E. coli* and its drug resistant strain, an NDM-1 carbapenemase isolate that is resistant to many of the major classes of antibiotics, with MIC values of 3 μg/mL. The growth of clinical isolated strains, *Streptococcus pyogenes* and *S. agalactiae* were inhibited with MIC values of 1.5 μg/mL. *M. catarrhalis* ATCC 25240 and *P. aeruginosa* ATCC BAA-2108 were inhibited with MIC values of 3 μg/mL and 6 μg/mL, respectively. In addition, **1** also showed a moderate antibacterial activity against *Klebsiella pneumoniae* and *Acinetobacter baumannii* strains with MIC values of 48 μg/mL, which are close to that of ceftazidime (**Table 1**).

**Table 1:** Minimum inhibitory concentrations of 1 against a panel of bacterial strains.

Organism	MIC (μg/mL)		
	1	Methicillin	Ceftazidime
*B. subtilis* 168	1.5	<1	3
*S. aureus* ATCC 29213	1.5	<1	6
*S. aureus* ATCC 29247	1.5	6	12
*S. aureus* ATCC 33591^a^	3	1024	>48
*S. aureus* ATCC 33592^a^	1.5	512	>48
*S. aureus* ATCC 43300^a^	1.5	512	48
*S. aureus* ATCC BAA-41^a^	3	1024	>48
*S. aureus* ATCC BAA-1717^a^	3	512	>48
*S. aureus* ATCC BAA-1720^a^	3	1024	>48
*S. aureus* ATCC BAA-1747^a^	3	256	48
*S. aureus* USA300 #417^a^	3	512	>48
*S. aureus* USA300 #2690^a^	3	256	48
*S. epidermidis* ATCC 12228	0.75	0.75	1.5
*E. faecalis* ATCC 29212	0.75	0.75	1.5
*E. faecium* ATCC 49624	0.75	1.5	3
*E. faecium* ATCC 700221^b^	1.5	1.5	3
*E. coli* ATCC 25922	3	3	3
*E. coli* ATCC BAA-2469^c^	3	>1024	>48
*S. pyogenes* ATCC 12344	1.5	ND	<1.5
*S. pyogenes* ATCC 19615	1.5	ND	<1.5
*S. agalactiae* ATCC 13813	1.5	ND	1.5
*M. catarrhalis* ATCC 25240	3	ND	<1.5
*P. aeruginosa* ATCC BAA-2108^d^	6	>256	6
*K. pneumoniae* ATCC BAA-1144^e^	48	>256	48
*A. baumannii* ATCC 19606	48	>256	24

*Methicillin was used as a positive control in this test. ^a^These strains are MRSA; ^b^A vancomycin-resistant strain, MIC of vancomycin is higher than 96 μg/mL (Sun et al., 2014); ^c^A NDM-1 expressing strain, which is resistant to most of the antibiotics, except tigecycline and colistin; ^d^An multidrug resistant strain; ^e^An AmpC beta-lactamase expressing strain. ND = not detect.*

### Time-Killing Curve Determinations

**FIGURE 3:**
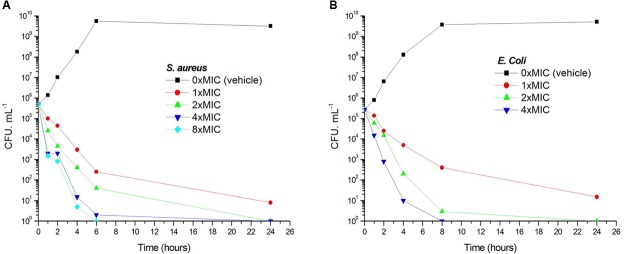
**Time-killing curve and bacterial survival curve for 1.** At time zero, samples of a growing culture of *S. aureus* ATCC 29213 were incubated with concentrations of 1 equivalent to 1× (red), 2× (green), 4× (blue), or 8× (cyan) the MIC **(A)**. Samples of a growing culture of *E. coli* ATCC 25922 were incubated with concentrations of 1 equivalent to 1× (red), 2× (green), or 4× (blue) the MIC **(B)**. Vehicle (1% DMSO; black) was included. Samples were removed at the time intervals indicated for the determination of viable cell counts.

Viable counts for the determination of killing curves were performed as previously described to investigate whether the antibacterial activity of **1** is bactericidal or not (Wikler et al., 2009). Sample killing curves resulting from **1** against *S. aureus* ATCC 29213 and *E. coli* ATCC 25922 are presented in **Figure 3**. The control showed no reduction in the counts of CFU from control inoculum. **Figure 3A** showed that **1** at 1× MIC concentration caused a reduction of 1 × 10^2^ CFU mL^-1^ for *S. aureus* in 4 h and to below the lowest detectable limit (10^3^ CFU mL^-1^) in 24 h. In the *E. coli* bacterial survival assay, 4× MIC of **1** can rapidly reduce the viable counts below the lowest detectable limit after 4 h incubation, and the counts at MIC concentration were maintained under the lowest detectable limit for over 24 h (**Figure 3B**). The results revealed that the antibacterial activity was consistent with a bactericidal mode of action.

### Compound 1 Disrupts FtsZ Function *In Vitro*

**FIGURE 4:**
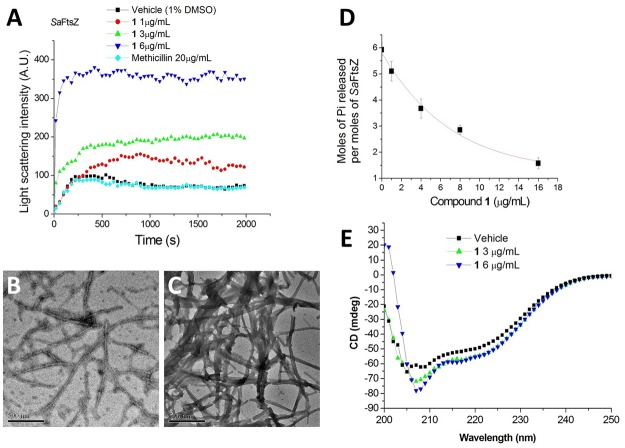
**Impact of 1 on the polymerization and GTPase activity of *S. aureus* FtsZ. (A)** Time-dependent polymerization profiles of *S. aureus* FtsZ in the absence and presence of **1** at a concentration range from 1 to 6 μg/mL. **(B,C)** Electron micrographs of *S. aureus* FtsZ polymers in the absence **(B)** and in the presence **(C)** of 3 μg/mL of **1**. Scale Bar = 500 nm. **(D)** The amount of Pi released per mol *Sa*FtsZ in the absence and presence of various concentrations of **1**. **(E)** The CD spectra of FtsZ in the absence and presence of **1** at a concentration range from 3 to 6 μg/mL.

It is interesting to note that similar cell filamentation phenomena were also found with FtsZ inhibitors from different chemotypes, such as 9-phenoxyalkyl substituted berberine derivatives and PC190723 (Haydon et al., 2008; Sun et al., 2014), suggesting that **1**-induced cell elongation may be attributed to the **1**-FtsZ interaction. To confirm whether this compound is able to disturb FtsZ activity *in vitro*, *S. aureus* FtsZ and *E. coli* FtsZ were cloned, overexpressed, and purified, and some biochemical assays were employed. As a first step to validate that FtsZ is the target of **1**, we assessed the impact of **1** on the polymerization dynamics of FtsZ using 90° light scattering in a thermostatically (37°C) controlled fluorescence spectrometer in which changes in FtsZ polymerization are reflected by corresponding changes in absorbance at 600 nm (Chan et al., 2015). **Figure 4A** shows the time-dependent polymerization profiles of *Sa*FtsZ in the absence and presence of **1** at a concentration range from 1 to 6 μg/mL. The similar phenomena can be observed in the time-dependent polymerization profiles of *Ec*FtsZ with **1** (Supplementary Figure S1). These results prove that **1** stimulates FtsZ polymerization in a concentration-dependent manner, which is similar to that of some FtsZ-targeting compounds (Andreu et al., 2010; Kaul et al., 2012, 2013b, 2015; Kelley et al., 2012). Methicillin is also included in the assay as a non-FtsZ-targeting control antibiotic and, as expected, it does not affect FtsZ polymerization. The effect of **1** on FtsZ polymer formation was also visualized using transmission electron microscopy (TEM) (Kaul et al., 2012). It was found that the size and thickness of the FtsZ polymers and the bundling of the FtsZ protofilaments were drastically increased with **1** (**Figures 4B,C**). At 3 μg/mL of **1**, the thickness of FtsZ protofilaments was found to have increased from ∼10 nm to ∼90 nm (**Figure 4C**). We further tested the inhibitory effect of **1** on the GTPase activity of FtsZ. The effects of various concentrations of **1** on the GTPase activity of *Sa*FtsZ or *Ec*FtsZ were estimated after 30 min of FtsZ assembly. The results shown that **1** decreased the rate of GTP hydrolysis of FtsZ in a concentration-dependent fashion (**Figure 4D** and Supplementary Figure S2). For example, 1, 4, and 8 μg/mL of **1** inhibited GTPase activity of *Sa*FtsZ by 14, 38, and 58%, respectively (**Figure 4D**). The results indicate that **1** inhibits the rate of GTP hydrolysis of FtsZ may due to modulating the assembly of FtsZ. Apart from polymerization and GTPase assays, the effects of **1** on the secondary structures of FtsZ were determined by monitoring far-UV CD spectra of FtsZ (**Figure 4E**). The results revealed that **1** can significantly perturb the secondary structures of FtsZ. By analysis of the CD spectra using Yang’s reference, it indicated that the secondary structure of FtsZ contained ∼31.5% α-helix, ∼21.1% β-sheet, and ∼47.4% other structures. When using 6 μg/mL of **1**, α-helix increased to ∼37.6% and β-sheet decreased to 14.2%. These confirmation changes may also be the cause of disruption of FtsZ function.

### Effect of Compound 1 on the Z-Ring Formation

**FIGURE 5:**
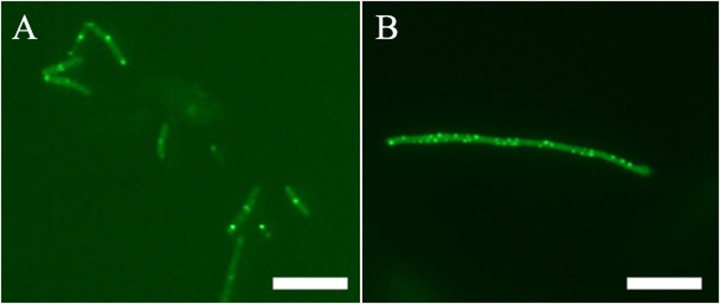
**Effect of Z-ring formation of 1 on *B. subtilis*.** Bacteria were grown in the absence **(A)**, and presence of 1 μg/mL of **1 (B)**. Scale Bar = 10 μm.

In addition to *in vitro* study, on-target effect of **1** in live bacteria is also important. A green fluorescent protein (GFP)-FtsZ constructed in *B. subtilis* 168 was used to monitor the impact of the compound on the formation of FtsZ Z-rings (Stokes et al., 2005). We treated the bacteria with DMSO (solvent control), or **1** and examined the bacteria using fluorescence microscopy. In the absence of compound, fluorescent foci corresponding to Z-rings are evident at midcell (**Figure 5A**). In contrast, the **1**-treated bacteria lack these midcell foci, and FtsZ was found to distribute as discrete and punctate foci throughout the elongated cell, indicating mislocalization of the FtsZ protein (**Figure 5B**). As **1** enhances FtsZ polymerization, it is likely that those discrete and punctate fluorescent foci in **1**-treated bacteria are multiple non-functional FtsZ polymeric structures. Observation of such disorganized FtsZ in elongated bacterial cells is typical of agents that disrupt FtsZ polymerization (Hurley et al., 2016).

### Predicted Binding Mode of Compound 1

**FIGURE 6:**
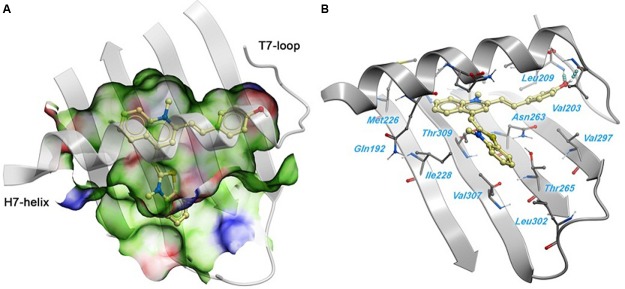
**Predicted binding mode of 1 against the *S. aureus* FtsZ. (A)** Binding site surface colored by ligand binding properties (green = hydrophobic, red = hydrogen bond acceptor propensity, blue = hydrogen bond donor propensity). The predicted binding mode of compound 1 is shown in a “ball and sticks” representation (light yellow = carbon, blue = nitrogen, red = oxygen, yellow = sulfur, white = polar hydrogen). **(B)** FtsZ residues in close contact with 1 are labeled and shown in a “ball and sticks” representation. Hydrogen bonds are represented with blue spheres.

A 2.01 Å crystal structure of *S. aureus* FtsZ apo-form (Tan et al., 2012) was used to identify a potential binding site for **1**. The highest docking score positioned the ligands bind near the T7-loop and H7-helix. Since the binding site is a relatively narrow cleft delimited by the H7-helix, the T7-loop, and a four-stranded β-sheet, the substrate requires some degree of planarity in its structure to fit in. The docking result suggested a large number of favorable hydrophobic interactions between the molecule and the side chains of Leu200, Val203, Leu209, Met226, Ile228, Leu261, Val297, Leu302, Val307, and Ile311. Moreover, the hydroxyl group of **1** was predicted to participate in two hydrogen bonds with the backbone carbonyl of Val203 and the backbone amide of Leu209 (**Figure 6**).

### Compound 1 Exerts Little or no Impact on the Mammalian Tubulin

**FIGURE 7:**
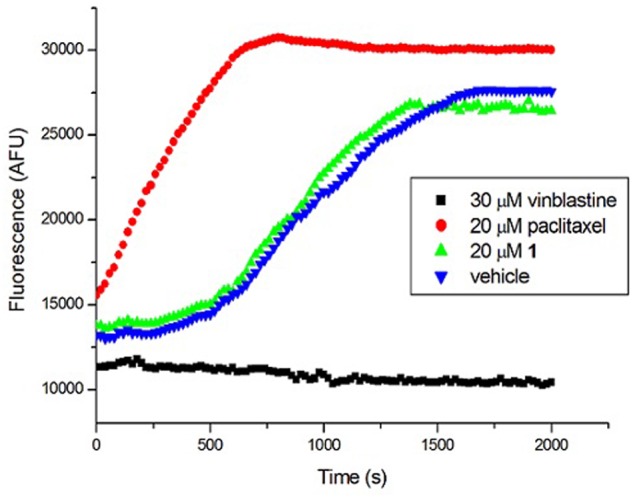
**Effect of 1 on the polymerization of mammalian tubulin.** (Blue) Tubulin was treated with 1% DMSO; (Green) Tubulin was treated with 20 μM of **1**; (Red) Tubulin polymerization was enhanced by 20 μM of paclitaxel; (Black) Tubulin polymerization was inhibited by 30 μM of vinblastine.

Tubulin is the closest mammalian functional homolog to bacterial FtsZ. We therefore sought to determine whether **1** would exert a similar effect on mammalian tubulin because our results (**Figure 4**) indicated that the compound is a potent stimulator of FtsZ polymerization. In the presence of vinblastine (30 μM), a tubulin inhibitor, the polymerization of mammalian tubulin was completely inhibited. On the opposite, the fluorescence intensity was significantly increased in the presence of paclitaxel (20 μM), confirming that the rate of polymerization was significantly enhanced. **1** (20 μM) showed similar results to a control experiment with 2% DMSO-treated mammalian tubulin, indicating that **1** is not a stimulator/inhibitor of tubulin polymerization (**Figure 7**).

### Cytotoxicity of Compound 1

To probe for any potential mammalian cytotoxicity, we used a MTS tetrazolium assay to assess the cytotoxicity of **1** against two mammalian cell lines (L929, HK-2). **1** was found to be minimally toxic to these cell types, with 50% inhibitory concentrations (IC_50_s) higher than 50 μg/mL (**Table 2**), which are much higher than the MICs (0.75–3.0 μg/mL) of **1** against bacterial strains, indicating no significant toxicity toward normal mammalian cells.

**Table 2:** Cytotoxicity of 1 on mammalian cells.

IC_50_ against L929	IC_50_ against HK-2
96.5 μM (∼53 μg/mL)	98.15 μM (∼54 μg/mL)

### Antimicrobial Study of Compound 1 under Different Nutrient Conditions

**FIGURE 8:**
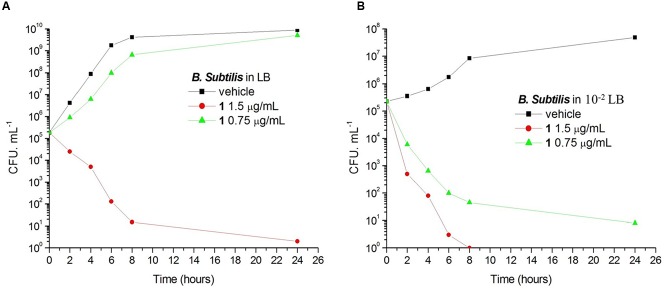
**The growth curves of *B. subtilis* 168 in LB and 10^-2^ LB for 1.** At time zero, samples of a growing culture of *B. subtilis* 168 in LB were incubated with **1** at concentrations of 1.5 μg/mL (red), or 0.75 μg/mL (green; **A**). Samples of a growing culture of *B. subtilis* 168 in 10^-2^ LB were incubated with **1** at concentrations of 1.5 μg/mL (red), or 0.75 μg/mL (green; **B**). Vehicle (1% DMSO; black) was included. Samples were removed at the time intervals indicated for the determination of viable cell counts.

**FIGURE 9:**
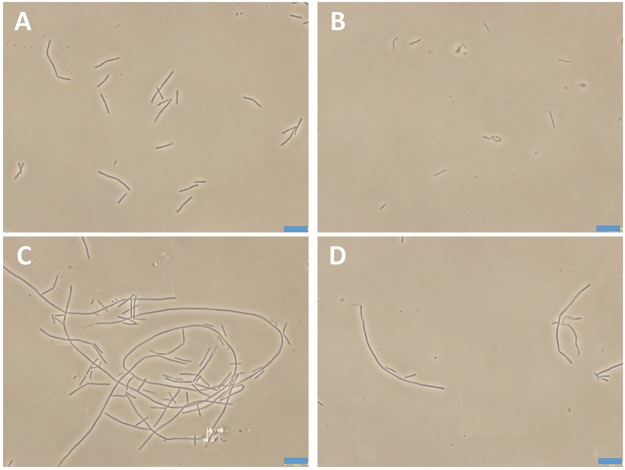
**The morphology of *B. subtilis* 168 in LB or 10^-2^ LB.** *B. subtilis* 168 cells cultured in LB for 24 h with absent **(A)** or 0.75 μg/mL of **1 (C)**. *B. subtilis* 168 cells cultured in 10^-2^ LB for 24 h with absent **(B)** or 0.75 μg/mL of **1 (D)**.

Bacteria change their size and growth rate when cultivated in nutrient poor medium (Chien et al., 2012; Mandal et al., 2013). It was reported that bacteria were more resistant to beta-lactam antibiotics in a rich medium than in a relatively poor medium (Wang et al., 2015). To further investigated the antimicrobial activity of Compound **1** under different nutrient conditions, we investigated the growth curves of *B. subtilis* 168 cells in LB or 10^-2^ LB medium with different concentrations of **1**. And also observed the morphology of *B. subtilis* growing in the two different nutrient conditions. The results indicated that *B. subtilis* cells cultured in a nutrient rich medium have a faster growth rate and longer morphology than that cultured in the nutrient poor medium (**Figures 8, 9**). **Figure 8A** showed that **1** at 1.5 μg/mL decreased the cell numbers of *B. subtilis* below the lowest detectable limit (10^3^ CFU mL^-1^) after incubation for 6 h; however, using 0.75 μg/mL of **1** cannot inhibit the growth of *B. subtilis* in LB. On the other hand, when *B. subtilis* cells were cultured in 10^-2^ LB, using 1.5 μg/mL of **1** can decreased the cell numbers to the lowest detectable limit after incubation for 4 h, and also using 0.75 μg/mL of **1** showed inhibitory effect on the growth of the cells (**Figure 8B**). These results indicated that **1** has a better antibacterial activity against bacteria cultured in the nutrient poor condition than that in the nutrient rich condition. These results may due to rapid growth cells with bigger size having much more FtsZ proteins than the slow growing cells in the nutrient poor medium (Chien et al., 2012). In addition, using 0.75 μg/mL of **1** can inhibit the cell division of *B. subtilis* in both of the culture media. And the phenomena of cell elongation were found sharper in LB than that of in 10^-2^ LB (**Figures 9C,D**).

## Discussions

In the present study, we have found that compound **1** induced filamentation in *B. subtilis* 168, showing that it inhibits bacterial cytokinesis. The antibacterial tests showed that **1** possessed strong and broad spectrum antibacterial activity. **1** was also found to bind to FtsZ *in vitro*; it enhanced the assembly and bundling of FtsZ protofilaments and decreased the GTPase activity of FtsZ. In addition, sequencing data of **1**-induced resistant mutants revealed that this compound inhibit cell division by disturbing the Z-ring formation through a direct interaction with FtsZ.

**1** increased the light scattering intensity of FtsZ protofilaments, indicating that it enhanced the polymerized mass of FtsZ protofilaments and/or enhanced the bundling of FtsZ protofilaments (**Figure 4**). Electron microscopic analysis of the polymerization reaction showed that **1** strongly increased the length of FtsZ protofilaments and the extent of bundling of the protofilaments (**Figures 4B,C**). The increase in the polymerization and bundling of FtsZ may be due to the conformational switch to the high-affinity state that enables polymer assembly (Elsen et al., 2012). The similar enhancement of FtsZ polymerization can also be found in some of FtsZ-targeting compounds, such as guanidinomethyl biaryl derivatives, dimethoxyisoquinoline derivatives, PC190723 and its analogs (Andreu et al., 2010; Kaul et al., 2012, 2013b, 2015; Kelley et al., 2012). The assembly dynamics of FtsZ is considered to be regulated by the GTPase activity of FtsZ (Margolin, 2005). Some FtsZ inhibitors which can enhance the FtsZ polymerization, such as PC190723 and Z3, were reported to inhibit the GTPase activity of FtsZ (Margalit et al., 2004; Haydon et al., 2008; Andreu et al., 2010). In this project, **1** was also found to decrease the GTPase activity of FtsZ in a dose dependent manner (**Figure 4D** and Supplementary Figure S2). And around 58% inhibition of GTP hydrolysis was found in the presence of 8 μg/mL (∼15 μM) of compound **1**. Most of the FtsZ inhibitors have been reported to suppress the GTPase activity of FtsZ (Margalit et al., 2004; Chan et al., 2013; Sun et al., 2014; Hurley et al., 2016). For example, zantrins inhibited GTP hydrolysis with IC_50_ values ranging from 4 to 100 μM (Margalit et al., 2004). 50% inhibition of GTP hydrolysis was found in the presence of 37.8 μM of a 9-phenoxyalkylberberine derivative (Sun et al., 2014). Similarly, quinuclidine and its analogs inhibited the GTPase activity of *Sa*FtsZ with an IC_50_ value of 37.5 μM (Chan et al., 2013). Thus the inhibitory effect of **1** on GTPase activity was comparable with that of other known FtsZ inhibitors. **1** was also found to disturb the secondary structure of FtsZ by monitoring CD spectra of FtsZ. Curcumin known as a FtsZ inhibitor also showed similar effect on FtsZ (Rai et al., 2008). The changes of secondary structure may be the cause of disruption of FtsZ function. Tubulin is the closest mammalian functional homolog to bacterial FtsZ. Twenty micrometer of **1** does not disturb the polymerization of tubulin. For the FtsZ protein, 3 μg/mL (∼6 μM) of **1** can effectively enhance FtsZ polymerization (**Figure 4A** and Supplementary Figure S1). These results suggest that **1** is a specific enhancer of bacterial FtsZ polymerization without any effect on mammalian tubulin.

**1** can strongly inhibit the cell proliferation against all the tested Gram-positive strains (including MRSA and VREF) with MIC values from 0.75 to 3 μg/mL. And the inhibitory effects of 1 against these strains were slightly better or similar to that of some reported FtsZ inhibitors. For Example, 9-phenoxyalkylberberine derivative 2 inhibited the cell division with MIC values from 2 to 8 μg/mL against MRSA and VREF (Sun et al., 2014). Quinuclidine 1 was found to inhibit the growth of S. aureus with a MIC value of 24 μg/mL (Chan et al., 2015). PC190723 inhibited the proliferation of *S. aureus* strains at ∼1 μg/mL (Haydon et al., 2008). In the other hand, **1** also showed strong inhibitory effects against most of the tested Gram-negative strains (MIC values from 1.5 to 6 μg/mL, **Table 1**). To date, there is only a few FtsZ inhibitors reported to have potent or moderate anti-Gram-negative bacterial activity. For instance, 9-phenoxyalkylberberine derivative 2 and Quinuclidine 1 inhibited the growth of *E. coli* with MIC values of 32 μg/mL (Sun et al., 2014; Chan et al., 2015). Guanidinomethyl biaryl derivative 13 has MIC values from 2 to 32 μg/mL against the Gram-negative strains (Kaul et al., 2012). Compared to the inhibitory effects against Gram-negative strains, **1** have much stronger activities than that of reported FtsZ inhibitors, which could be ascribed to this compound’s ability to pass through the outer membrane of gram-negative bacteria (Keffer et al., 2013; Kaul et al., 2014).

## Conclusion

In summary, we identified a new small molecule which is able to disrupt FtsZ dynamic specifically. This compound effectively enhances FtsZ protein assembly but not tubulin polymerization, blocks bacterial cytokinesis, and eventually impairs bacterial cell division. Furthermore, **1** displays potent antibacterial activity against drug-resistant pathogenic bacteria, including MRSA, VREF and NDM-1 expressing *E. coli*. These merit characteristics of the compound coupled with minimal cytotoxicity to mammalian cells make it be an attractive hit for the design of innovative compounds that enhance specificity target to FtsZ and have stronger antibacterial activities. Several modifications on the structure of this thiazole orange derivative are currently being made in our laboratories.

## Author Contributions

Conceived and designed the experiments: NS, Y-CL, and K-YW. Performed the experiments: NS, Y-JL, F-YC, R-LD, and Y-yZ. Analyzed the data: NS, Y-JL, and F-YC. Contributed reagents/materials/analysis tools: L-YS, KZ, RA, and CZ. Wrote the paper: NS, Y-JL, and K-YW.

## Conflict of Interest Statement

The authors declare that the research was conducted in the absence of any commercial or financial relationships that could be construed as a potential conflict of interest.
