# Supplementary material for: A Thiazole Orange Derivative Targeting the Bacterial Protein FtsZ Shows Potent Antibacterial Activity
Source: Front Microbiol. 2017 May 11;8:855. doi: 10.3389/fmicb.2017.00855 (PMC5426085; doi:10.3389/fmicb.2017.00855)
Supplement: Supplementary file 1 [file Data_Sheet_1.PDF]

Supplementary Material

A thiazole orange derivative targeting the bacterial protein FtsZ shows high potent antibacterial activity

Ning Sun[1], Yu-Jing Lu[2,*], Fung-Yi Chan[1], Ruo-Lan Du[1], Yuan-yuan Zheng[2], Kun Zhang[2], Lok-Yan So[1], Ruben Abagyan[3], Chao Zhuo[4] Yun-Chung Leung[1], Kwok-Yin Wong[1,*]

[1.] Department of Applied Biology and Chemical Technology and the State Key Laboratory of Chirosciences, The Hong Kong Polytechnic University, Kowloon, Hong Kong SAR, P.R. China.
[2.] Institute of Natural Medicine and Green Chemistry, School of Chemical Engineering and Light Industry, Guangdong University of Technology, Guangzhou 510006, P.R. China;
[3.] Skaggs School of Pharmacy & Pharmaceutical Sciences, University of California San Diego, La Jolla, California, United States of America.
[4.] State Key Laboratory of Respiratory Diseases, the First Affiliated Hospital of Guangzhou Medical University, Guangzhou, P.R. China.

*Correspondence:
Kwok-Yin Wong kwok-yin.wong@polyu.edu.hk;
Yu-Jing Lu luyj@gdut.edu.cn

List of contents:

1. Impact of **1** on the polymerization of *E.coli* FtsZ
2. Impact of **1** on the GTPase activity of *E.coli* FtsZ
3. Synthesis and characterization of **1**
References

1. Impact of 1 on the polymerization of *E.coli* FtsZ

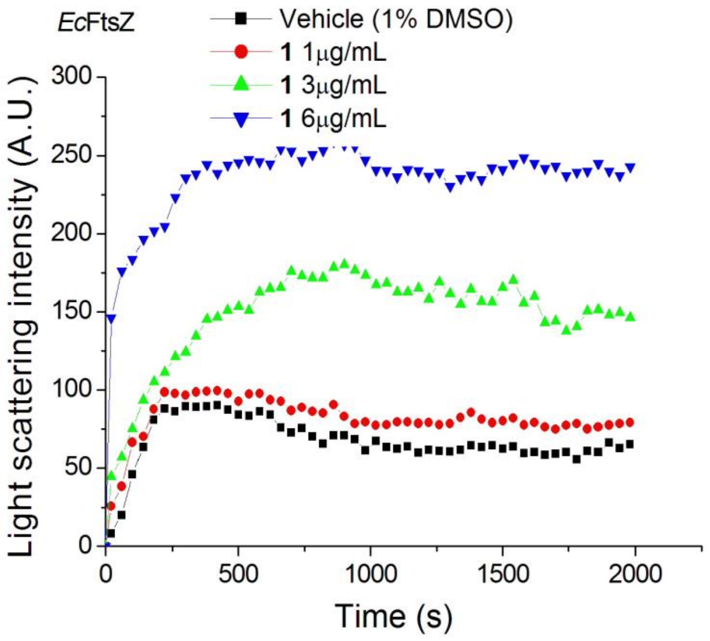

Figure S1. Time-dependent polymerization profiles of *E.coli* FtsZ in the absence and presence of **1** at a concentration range from 1 to 6 μg/mL.

2. Impact of **1** on the GTPase activity of *E.coli* FtsZ

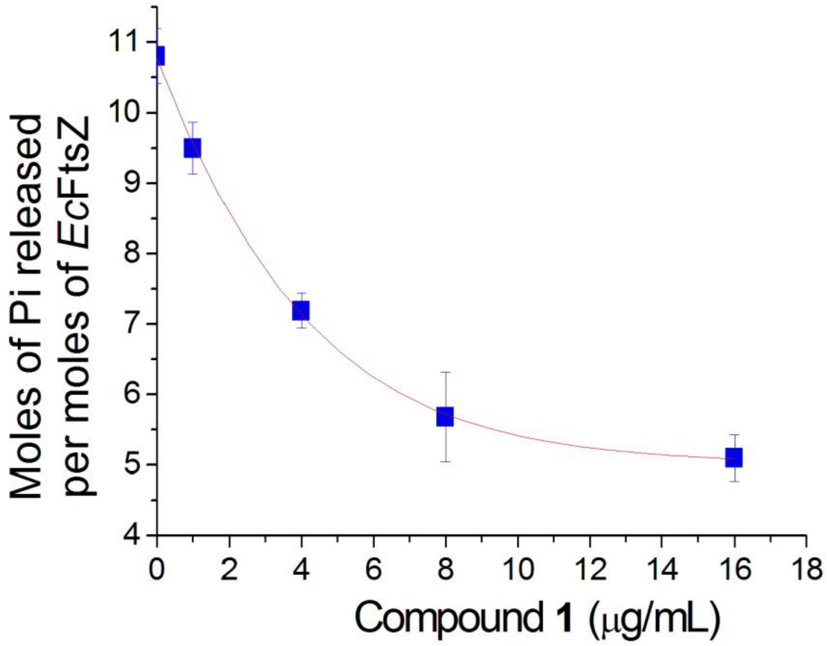

Figure S2. The amount of Pi released per mol *Ec*FtsZ in the absence and presence of various concentrations of **1**.

3. Synthesis and characterization of **1**

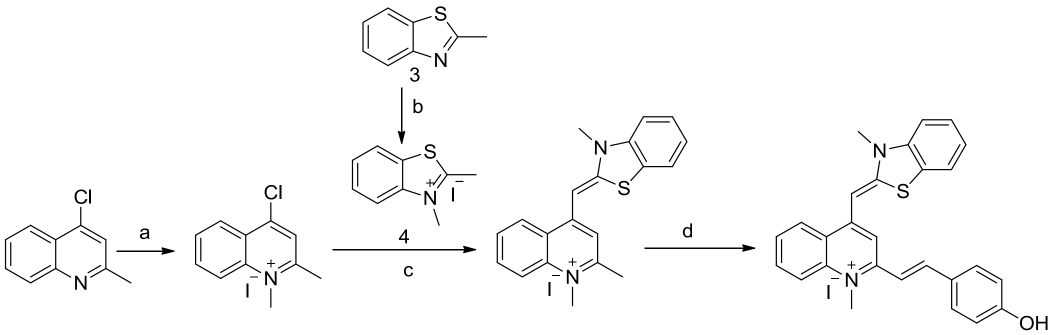

Scheme S1. Synthesis route of **1**. Reagents and conditions[1]: (a) iodomethane, tetramethylene sulfone, 52 ℃, reflux; (b) iodomethane, absolute ethanol, 80 ℃, reflux; (c) $NaHCO_3$, methanol, room temperature; (d) 4-hydroxybenzaldehyde, 4-methylpiperidine, n-butanol, 135 ℃, reflux. (All the reagents were purchased from Sigma.)

^{1}H and ^{13}C NMR spectra were recorded using TMS as the internal standard in DMSO-d_6 with a Bruker BioSpin GmbH spectrometer at 400 MHz, respectively. Mass spectra (MS) were recorded on Bruker amaZon SL mass spectrometer with an ESI or ACPI mass selective detector and high resolution mass spectra (HRMS) were recorded on Shimadzu LCMS-ITTOF. **1** was identified by HPLC (Shimadzu Technologies) by using a C18 column (4.6×150mm) with a 4 min Elution and a gradient of 5%–95% CH_3CN -H_2O (containing 0.5% acetic acid). The HPLC contained a UV detector at λ = 250nm, 350 nm, 430 nm, and 500 nm.

1: Purple solid; m.p. = 275-281°C; ^{1}H NMR (400 MHz, DMSO) δ 8.59 (t, J = 17.8 Hz, 1H), 8.04–7.82 (m, 3H), 7.79–7.60 (m, 3H), 7.49 (d, J = 22.2 Hz, 3H), 7.36 (t, J = 18.0 Hz, 3H), 6.87 (d, J = 7.9 Hz, 2H), 6.68 (s, 1H), 3.98 (d, J = 22.1 Hz, 3H), 3.83 (s, 3H). 13C NMR (100 MHz, DMSO) δ 160.39 (s), 159.11 (s), 152.57 (s), 147.55 (s), 141.65 (s), 140.78 (s), 139.26 (s), 133.45 (s), 131.04 (s), 128.37 (s), 126.71 (s), 125.49 (s), 124.38 (s), 124.09 (s), 123.73 (s), 123.32 (s), 118.79 (s), 117.93 (s), 116.25 (s), 112.79 (s), 107.92 (s), 87.81 (s), 38.36 (s), 33.99 (s).HRMS (ESI): m/z calcd for $C_{27}H_{23}N_2OS^+$ ($[M-I]^+$) 423.1530; found 423.1521.The HPLC retention time of **1** was 2.18 min.

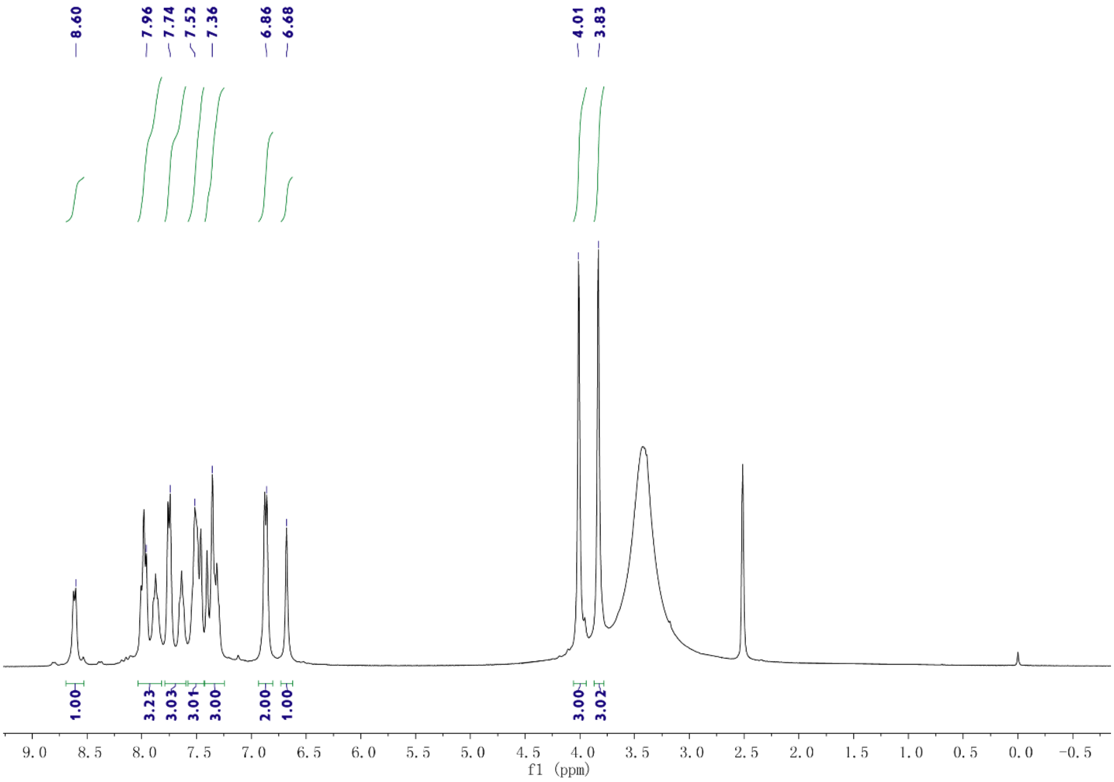

Figure S3. ^{1}H NMR spectrum of **1**

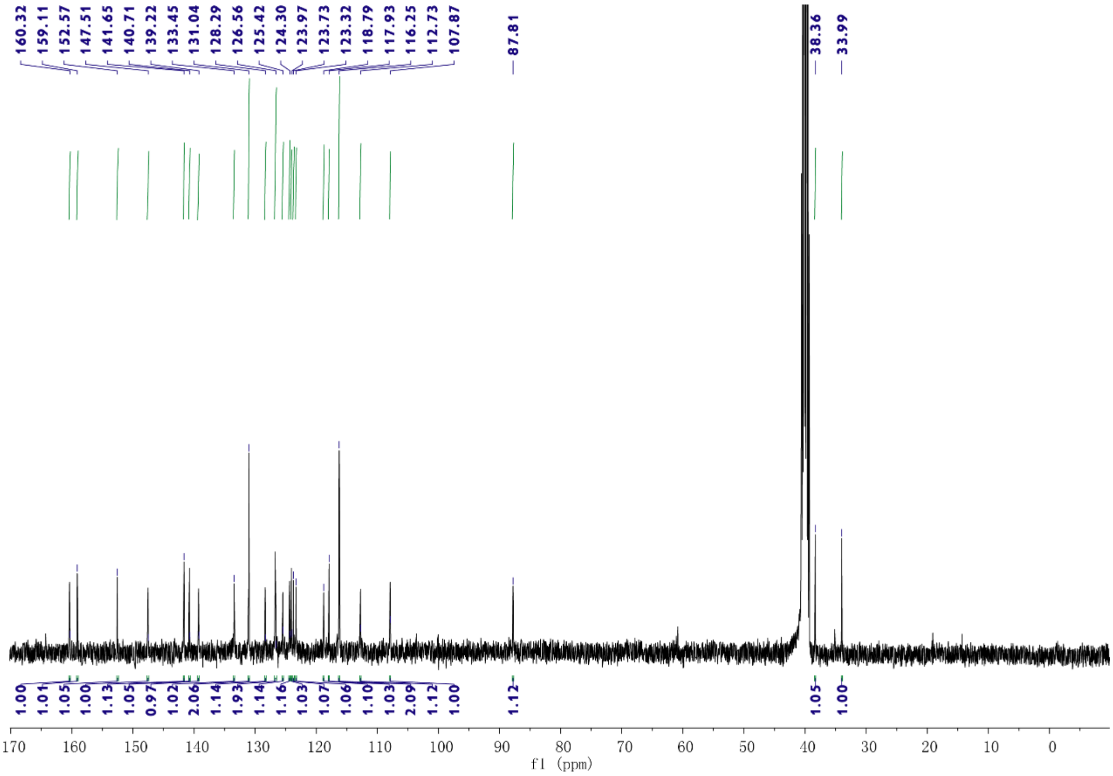

Figure S4. ^{13}C NMR spectrum of **1**

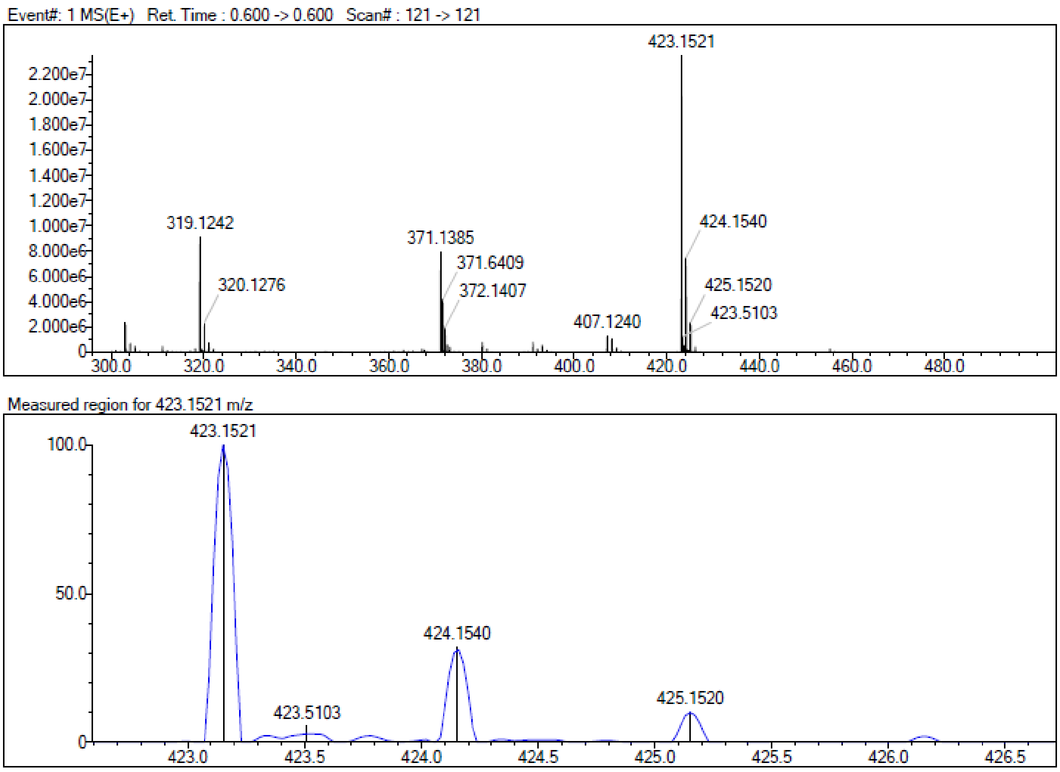

Figure S5. MS analysis of **1**

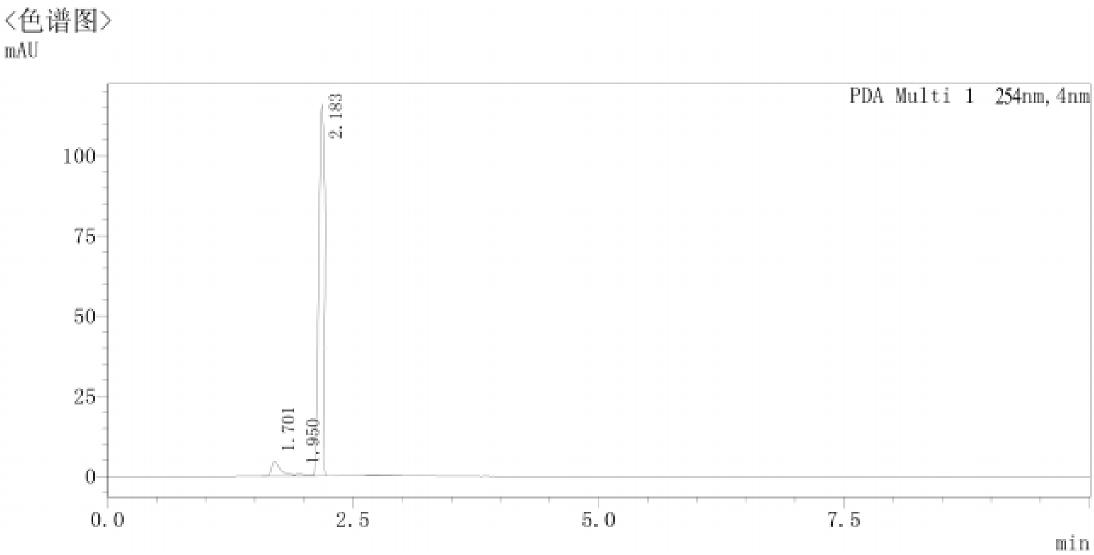

Figure S6. HPLC analysis of **1.**

Reference

1. Lu YJ, Deng Q, Hu DP, Wang ZY, Huang BH, et al. (2015) A molecular fluorescent dye for specific staining and imaging of RNA in live cells: a novel ligand integration from classical thiazole orange and styryl compounds. Chem Commun (Camb) 51: 15241-15244.
